# Jingtian granule alleviates adenine-induced renal fibrosis in mice through SIRT3-Mediated deacetylation of P53

**DOI:** 10.3389/fphar.2025.1526414

**Published:** 2025-03-12

**Authors:** Zhili Xiong, Xinyu Hu, Rui Wang, Chengyin Li, Huanbo Cheng, Wei Zhao, Yinfeng Shen, Linqun Wang, Weinan Li, Xiaoyun Zhu, Yuanming Ba

**Affiliations:** ^1^ Hubei University of Chinese Medicine, Wuhan, China; ^2^ Hubei Provincial Hospital of Traditional Chinese Medicine, Wuhan, China; ^3^ Hubei Sizhen Laboratory, Wuhan, China; ^4^ Affiliated Hospital of Hubei University of Traditional Chinese Medicine, Wuhan, China

**Keywords:** Jingtian granule (JT), chronic kidney disease (CKD), sirt3, ferroptosis, p53

## Abstract

**Background:**

Renal fibrosis is a hallmark and the final outcome of chronic kidney disease (CKD). Jingtian Granule (JT), a traditional formula used in the clinical treatment of CKD for many years. However, the mechanism of action of JT against renal interstitial fibrosis remain unknown.

**Objective:**

This study aimed to explore the potential effects and mechanisms of JT on adenine - diet - induced CKD in mice.

**Methods:**

Renal interstitial fibrosis was induced in mice by adenine - diet and treated with JT. Renal function was assessed by measuring blood urea nitrogen and serum creatinine levels. Masson’s staining and type I collagen expression were used to evaluate renal collagen deposition. RNA sequencing was used to analyze the expression levels of mRNA in mouse kidney samples after JT treatment. The levels of glutathione (GSH) and malondialdehyde (MDA) were measured to assess lipid peroxidation in the kidneys. Iron metabolism levels were detected by Prussian blue staining and measurement of iron content. The protein levels of SIRT3, P53, glutathione peroxidase 4 (GPX4), and solute carrier family 7 member 11 (SLC7A11) were detected by Western blot. Subsequently, under the premise of SIRT3 knockout, renal function, fibrosis level, iron metabolism level, and lipid peroxidation level were detected, and mitochondrial damage was observed by transmission electron microscope (TEM). In addition, human proximal tubule epithelial cells (HK - 2) were treated with Erastin to induce ferroptosis, followed by exposure to JT. The levels of reactive oxygen species (ROS) were detected.

**Results:**

JT significantly reduced collagen deposition in the kidneys. RNA sequencing identified 20 mRNAs that were differentially expressed in response to JT treatment. Bioinformatics analysis revealed that SIRT3 was a key mRNA regulated by JT. JT activated SIRT3 in fibrotic kidneys to inhibit the acetylation of P53. Under the premise of SIRT3 knockout, JT did not show significant therapeutic effects in inhibiting ferroptosis and fibrosis. *In vitro* experiments also showed that JT promoted the downregulation of ROS.

**Conclusion:**

SIRT3 is the key ferroptosis - related mRNA regulated by JT. The ability of JT to modulate the SIRT3/P53 signaling pathway may be a viable approach for the treatment of renal interstitial fibrosis.

## 1 Introduction

Chronic kidney disease (CKD) is a progressively worsening condition in which the kidneys fail to effectively filter waste and excess fluids from the blood. The primary causes of CKD include diabetes, hypertension, chronic nephritis, and polycystic kidney disease (Noble and Taal, 2019). Early symptoms are usually not noticeable. However, as the disease progresses, patients may experience edema, nausea, and itching. CKD has become a significant global health burden, especially in developing countries. According to the Global Burden of Disease Study, the incidence and mortality rates of CKD increased significantly from 1990 to 2016 (Xie et al., 2018). The global average prevalence of CKD is approximately 13.4%, with the highest rates in the Asia–Pacific region (Liyanage et al., 2022). Developing countries face a heavy burden due to a lack of medical resources and chronic disease management systems (Nugent et al., 2011).

Treatments for CKD are currently limited. One major treatment method involves blocking the renin‒angiotensin system (RAS) via drugs such as ACE inhibitors and ARBs. These drugs can effectively lower blood pressure and reduce proteinuria, slowing CKD progression. However, common side effects, such as hyperkalemia and hypotension, remain unavoidable obstacles (Stevens et al., 2013). Dialysis or kidney transplantation are the only expensive forms of renal replacement therapy that can be used to treat end-stage renal failure. According to the National Institutes of Health, end-stage renal failure has been documented in 1% of patients receiving medical care but accounts for 7% of the total healthcare budget (Foley and Collins, 2007). Therefore, seeking safe and effective treatments is essential.

Traditional Chinese medicine (TCM) has a long history in treating CKD, emphasizing holistic regulation, syndrome differentiation, and personalized treatment. As research has progressed, many Chinese herbs, such as *Astragalus*, *Salvia*, and *Angelica* (Li et al., 2024; Wu et al., 2023; Yuan et al., 2022), have shown positive antifibrotic and renoprotective effects. Jingtian granule (JT) is a Chinese herbal metabolites modified from the classical formulas Wuling San and Dahuang Gancao Tang and recorded in the “Treatise on Cold Damage and Miscellaneous Diseases.” Owing to its safety and effectiveness, JT has been used clinically for CKD treatment for many years and has been granted a patent by the China National Intellectual Property Administration (NOZL 2022 1 1670906.5). Research has indicated that emodin, an important metabolite of JT, significantly alleviates renal fibrosis by regulating miRNA/target gene mechanisms. miR-490-3p has been identified as a new target to prevent HMGA2-dependent signaling pathway-related renal injury (Wang et al., 2023). Additionally, rhubarb has been shown to alleviate chronic renal failure by reshaping the gut microbiota, reducing plasma urea and creatinine levels, and alleviating renal fibrosis and inflammation, demonstrating its potential clinical application in renal disease treatment (Wang et al., 2022). However, given the multimetabolite, multitarget, and multipathway characteristics of JT, detailed research on its pharmacology and mechanisms is needed to further elucidate its role in combating renal fibrosis.

Ferroptosis, a type of programmed cell death characterized by iron overload and lipid peroxidation buildup, was recently shown to be closely associated with CKD. Changes in iron overload and ferroptosis markers are present in the kidney tissues of CKD patients. Iron chelators can reduce ferroptosis and improve renal function (J. Li et al., 2023). SIRT3 is a mitochondrial deacetylase involved in regulating mitochondrial function, oxidative stress, and metabolic homeostasis (Perico et al., 2016). Previous evidence suggests that SIRT3 can regulate tissue fibrosis by targeting the deacetylation of tumor protein 53 (P53), a well-known tumor suppressor gene that functions mainly by regulating the cell cycle and inducing apoptosis, and it plays an important role in ferroptosis. Based on these findings, we hypothesize that JT improves CKD symptoms, at least in part, by regulating the SIRT3/P53/glutathione peroxidase 4 (GPX4) axis. We tested this hypothesis by evaluating the effects of JT on SIRT3 and acetylated P53 levels in a mouse CKD model and assessed the related changes in the expression of GPX4/solute carrier family 7 member 11 (SLC7A11) in the tissues of SIRT3^−/−^ mice. *In vitro*, we induced ferroptosis in the human proximal tubular epithelial cell line HK-2 with erastin and then treated the cells with JT to observe changes in ferroptosis, reactive oxygen species (ROS) production, SIRT3 expression, and P53 acetylation (Carrà et al., 2020). In this study, C57BL/6 mice, SIRT3^−/−^ mice, and HK-2 cells were used to investigate the changes in and effects of JT on the kidneys and related physical and chemical indicators in CKD model mice, ultimately clarifying the possible mechanisms of action of JT in treating CKD.

## 2 Materials and methods

### 2.1 Reagents

“Type A extracts” (Heinrich et al., 2022) are botanicals, the extracts of which are listed in national or regional pharmacopoeias as active metabolites in botanical medicines (licensed, marketed, or registered medicines) for controlled medical use. JT is a type A extract and the daily dosage of JT is as follows:*Polygonatum cyrtonema[* Asparagaceae *, Rhizoma siccum]:*15 g*;Rhodiola crenulata[* Crassulaceae *, Radix et R. siccum]:*10 g*;Poria cocos[*Polyporaceae*;Sclerotium siccum];Polyporus umbellatus[Polyporus, S. siccum]:*10 g*;Alisma orientale[*Alismataceae*, Tuber siccum]:*10 g*;Cinnamomum cassia [* Lauraceae *, Ramulus siccus]:*5 g*;Glycyrrhiza uralensis[* Fabaceae *, Radix et R. siccum]:*3 g*;Rheum officinale[* Polygonaceae *, Radix et R. siccum]:*5 g*;Atractylodes macrocephala [* Asteraceae *, R. siccum]:*10 g*;Dioscorea nipponica [* Dioscoreaceae *, R. siccum]:*12 g. These samples were identified by Prof. Cheng Huanbo of Hubei University of Traditional Chinese Medicine (Table 1). The above drugs were used in accordance with the relevant provisions of the Chinese Pharmacopoeia, 2020 Edition (Part I). The voucher samples are stored at the Institute of Standardization, School of Pharmacy, Hubei University of Traditional Chinese Medicine. All of the plant names were checked at https://www.worldfloraonline.org. These botanical drugs met the relevant requirements of the Chinese Pharmacopoeia (Xu et al., 2021).

**TABLE 1:** JT drug composition.

Latin binomial name	Family	Genus	Chinese Pin Yin name	Medicinal parts	Botanical drugs(g)
*Polygonatum cyrtonema*	Asparagaceae	Polygonatum	Huang jing	*Rhizoma siccum*	15
*Rhodiola crenulata*	Crassulaceae	Rhodiola	Hong jing tian	*Radix et Rhizoma siccum*	10
*Poria cocos*	*Polyporaceae*	*Poria*	Fu ling	*Sclerotium siccum*	15
*Polyporus umbellatus*	*Polyporus*	*Polyporaceae*	Zhu ling	*Sclerotium siccum*	10
*Alisma orientale*	*Alismataceae*	*Alismataceae*	Ze xie	*Tuber siccum*	10
*Cinnamomum cassia*	Lauraceae	Cinnamomum	Gui zhi	*Ramulus siccus*	5
*Glycyrrhiza uralensis*	Fabaceae	Glycyrrhiza	Gan cao	*Radix et Rhizoma siccum*	3
*Rheum officinale*	Polygonaceae	*Rheum*	Da huang	*Radix et Rhizoma siccum*	5
*Atractylodes macrocephala*	Asteraceae	Atractylodes	Bai zhu	*Rhizoma siccum*	10
*Dioscorea nipponica*	Dioscoreaceae	Dioscorea	Chuan shan long	*Rhizoma siccum*	12

### 2.2 Drug preparation

JT was prepared according to the classical method described in “Shang Han Lun”. After soaking in six volumes of water for 30 minutes, the herbs were boiled for 45 minutes. This process was repeated with another six volumes of water for 30 min. The combined filtrate was concentrated under reduced pressure at 60°C to a relative density of 1.08–1.10 and then spray-dried to obtain a dry extract powder. The extraction yield, measured by vacuum drying, was 17.61%. The dry extract was stored at −20°C. Valsartan (VAL,Cat. No. H20051350; Lunan Better Pharmaceutical Co., Jinan, China) was used as a positive control.

### 2.3 Determination of active metabolites in JT based on UPLC fingerprinting

#### 2.3.1 Chromatographic conditions

Mobile phase: acetonitrile (A)–0.2% phosphoric acid solution (B); injection volume: 1 μL; flow rate: 0.2 mL/min; column temperature: 30°C; detection wavelength: 265 nm. Gradient elution: 0–3 min, 5%→8% A; 3–15 min, 8%→15% A; 15–50 min, 15%→26% A; 50–60 min, 26%→42% A; 60–68 min, 42%→44% A; 68–73 min, 44%→70% A; 73–76 min, 70%→5% A; 76–80 min, 5% A; 60–68 min, 42%→44% A; 68–73 min, 44%→70% A; 73–76 min, 70%→5% A; and 76–80 min, 5% A.

#### 2.3.2 Preparation of the test solution

The appropriate amount of Jingtian particles was obtained, weighed to 2 g, placed in a stoppered conical flask, supplemented with 20 mL of methanol, weighed, subjected to ultrasonic (power 250 W, frequency 45 kHz) treatment for 30 min, cooled and then weighed, with methanol used to compensate for the loss of mass. The solution was filtered with a 0.22 μm microporous filter membrane to produce the solution of the test material.

#### 2.3.3 Preparation of the control solution

Appropriate amounts of 5-hydroxymethylfurfural, *Rhodiola rosea* glycoside, chlorogenic acid, cinnamic acid, rhubarbic acid, ammonium glycyrrhizinate, rhubarbine, glycyrrhizic acid, rhubarb phenol, and rutabaga rhubarbine controls were weighed precisely, and methanol was added to prepare a certain concentration of the control solution. An appropriate amount of each control was added to a volumetric flask, and methanol was added to prepare concentrations of 49.00, 103.50, 26.52, 18.96, 76.96, 25.97, 12.62, 9.68, 13.22, and 20.16 μg/mL, respectively.

### 2.4 Drug testing

A Wayeal LC3600 series ultrahigh-performance liquid chromatograph (Anhui Wan Yi Technology Co., Ltd.) and a Luna^®^ Omega Polar C_18_ column (150 mm × 2.1 mm, 1.6 μm, Phenomenex, United States) were used. The 5-hydroxymethylfurfural control (Batch No. 111626-202316, purity: 98.4%), *R. rosea* glucoside control (batch No. 110818-202210, Purity: 99.7%), chlorogenic acid control (batch No. 110753-202119, purity: 96.3%), cinnamic acid control (batch No. 110786-202305, purity: 98.8%), ammonium glycyrrhizinate control (batch No. 110731-202122, purity: 94.4%), rhubaric acid control (batch No. 110751-202122, purity: 94.4%), Purity: 98.8%, ammonium glycyrrhizinate control (batch No. 110731-202122, purity: 94.4%), rhubarbic acid control (batch No. 110757-201607, purity: 96.0%), rhubarbine control (batch No. 110756-201913, purity: 96.0%), blycyrrhizic hypophosphoric acid control (batch No. 110723-202316, purity: 99.6%), rhubarb phenol control (batch no. 110796-201922, purity: 99.4%), and aloe rhubarbin control (batch no. 110795-201710, purity: 98.3%) were purchased from the China Academy of Food and Drug Administration. Acetonitrile and phosphoric acid were chromatographically pure, and the water was ultrapure water. Samples S1 to S10 of Jingtian granules were prepared in the laboratory, samples S11 to S13 were prepared by Jin Brand Holding Zhengtang Pharmaceutical Company Limited, and the tablets used were purchased from Hubei Chenmei Traditional Chinese Medicine Company Limited.

### 2.5 Mouse model

Male SIRT3^−/−^ mice provided by Saiye Gene Co. (Suzhou, China) were bred in the laboratory. The experiment involved nine 6–8-week-old specific pathogen-free (SPF) male mice weighing 20 ± 2 g (from the Hubei Provincial Center for Disease Control and Prevention, Wuhan, China). The mice were acclimatized for 1 week under a 12-h light/dark cycle (55% ± 5% humidity, 23°C ± 2°C) with free access to food and water. The mice were split into the following groups at random (n = 9): (1) the control group, which was fed a regular diet; (2) the CKD group, which was fed a 0.2% adenine diet (w/w); and (3) the JT groups (high/medium/low), which were fed a 0.2% adenine diet and administered different doses of JT granules by gavage. In accordance with the “Pharmacological Methodology of Traditional Chinese Medicine (Zhou et al., 2011)”, JT granules were administered by gavage at low (0.24 g/kg), medium (0.48 g/kg), and high (0.96 g/kg) doses, with a gavage volume of 10 mL/kg body weight. The control and model groups were administered an equal volume of deionized water by gavage. Interventions lasted 2 weeks. (4) The VAL group was fed a 0.2% adenine diet and administered VAL by gavage for 2 weeks. In accordance with previous methods (Liu et al., 2023), the VAL group was gavaged the drug at 10 times the clinical dose (0.025 g/kg). The experiment lasted 6 weeks. At the end of the experiment, all the mice were euthanized, and their kidneys and plasma were collected. The kidneys were rinsed with isotonic saline and dissected to obtain renal tissue, which was stored in liquid nitrogen for the RNA-seq analysis. For additional analyses, the remaining samples were stored at −80°C. The requirements of the Animal Care and Use Committee of Hubei University of Traditional Chinese Medicine were met when animal research was conducted (HUCMS202302007).

### 2.6 Immunohistochemistry (IHC)

Antigen retrieval was performed on kidney tissue sections from each group using the SP two-step method, and endogenous peroxidase activity was blocked according to the instructions of an immunohistochemistry kit. After the antibody incubation, the sections were observed and photographed using a microscope.

### 2.7 Western blotting

Kidney tissue was treated with RIPA buffer containing a protease inhibitor cocktail (Beyotime, Cat. No. P0013B) to extract total protein. The protein concentration was determined Using a BCA protein assay kit (Beyotime, Cat. No. P0011). Protein samples were separated on SDS‒PAGE gels and transferred to polyvinylidene fluoride (PVDF) membranes. After blocking for 1 hour, the membranes were incubated overnight at 4°C with primary antibodies against SIRT3, GPX4, SLC7A11, P53, ac-P53, type I collagen and GAPDH. The membranes were incubated with an enhanced chemiluminescence (ECL) protein detection kit after being incubated with HRP-conjugated secondary antibodies for 1.5 h.

### 2.8 Cell culture

Human proximal tubular epithelial cells (HK-2) (Servicebio, Cat. No. STCC10303P-1) were cultured in DMEM supplemented with 10% FBS and 1% penicillin/streptomycin in a humidified environment at 37°C with 5% CO_2_. The main reagents used were as follows: CCK-8 kit (Servicebio, Cat. No. G4103-1 ML); DMEM/F12 (Thermo Fisher, Cat. No. A4192002); fetal bovine serum (Thermo Fisher, Cat. No. A5669701); erastin (Beyotime, Cat. No. SC0224-5 mg); ferrostatin-1 (MCE, Cat. No. HY-100579); and an ROS fluorometric assay kit (Beyotime, Cat. No. E-BC-F005). Eighteen healthy adult male SD rats weighing 180–220 g (from the Hubei Provincial Center for Disease Control and Prevention, Wuhan, China) were selected and adaptively fed under standard laboratory conditions for 1 week. The rats were randomly divided into three groups (n = 6) and administered different doses of JT (0.24 g/kg, 0.48 g/kg, or 0.96 g/kg) once a day for seven consecutive days. One hour after the last administration, the rats were anesthetized, and blood was collected from the abdominal aorta. Serum was obtained by centrifugation, complement was inactivated in a water bath at 56°C for 30 min, and the serum was prepared and stored at −80°C. The optimal concentration of JT-containing serum for intervention in the HK-2 cell model of erastin-induced injury was determined via the CCK-8 method. The corresponding drugs were administered, and the cells were incubated at 37°C with 5% CO_2_ for 24 h and 48 h. The culture medium was then removed, and CCK-8 solution was added. The absorbance at 450 nm was detected after 2 h of incubation in an incubator, and HK-2 cell viability was calculated to obtain the growth inhibition rate. The groups included the blank control, erastin, Fer-1, and JT groups. The experimental setup for the cell seeding and CCK-8 assays was the same as that described above. HK-2 cell morphology was observed. HK-2 cells in each group were treated for 48 h. The cell morphology and growth status were observed under an inverted fluorescence microscope. ROS levels were detected using an assay kit. HK-2 cells were cultured in DMEM supplemented with 10% fetal bovine serum and incubated at 37°C with 5% CO_2_. Each group was treated with working solution (1–10 μmol/L) and incubated at 37°C in the dark for 30–60 min. Fluorescence detection was performed using a microplate reader at an excitation wavelength of 300 nm or 488 nm and an emission wavelength of 610 nm.

### 2.9 Transmission electron microscope

Fresh kidney tissues (1 mm³) were fixed sequentially in 2.5% glutaraldehyde for 24 h, followed by 1% osmium tetroxide for 2 h. The samples were then dehydrated through a graded series of ethanol and acetone. After dehydration, the samples were infiltrated with a mixture of acetone and propylene oxide. Each sample was embedded in resin and then sliced using an ultramicrotome. The sections were stained with uranyl acetate and lead citrate. The ultrastructure of podocytes and the number of mitochondria in the CKD model mice were observed under a transmission electron microscope (TEM) (HITACHI, Cat. No. HT770).

### 2.10 Statistical analysis

The experimental findings were statistically analyzed with GraphPad 8.0. Differences between two groups were analyzed using an unpaired two-tailed Student’s t-test. The results are shown as $\bar{x}$ ± s. One-way analysis of variance (ANOVA) was used when the variance was homogenous and the data were normally distributed. Nonparametric tests were utilized when the variance was not homogenous or when the data did not follow a normal distribution. Statistics were deemed significant if *P* < 0.05.

## 3 Results

### 3.1 The analysis results of active metabolites in JT

**FIGURE 1:**
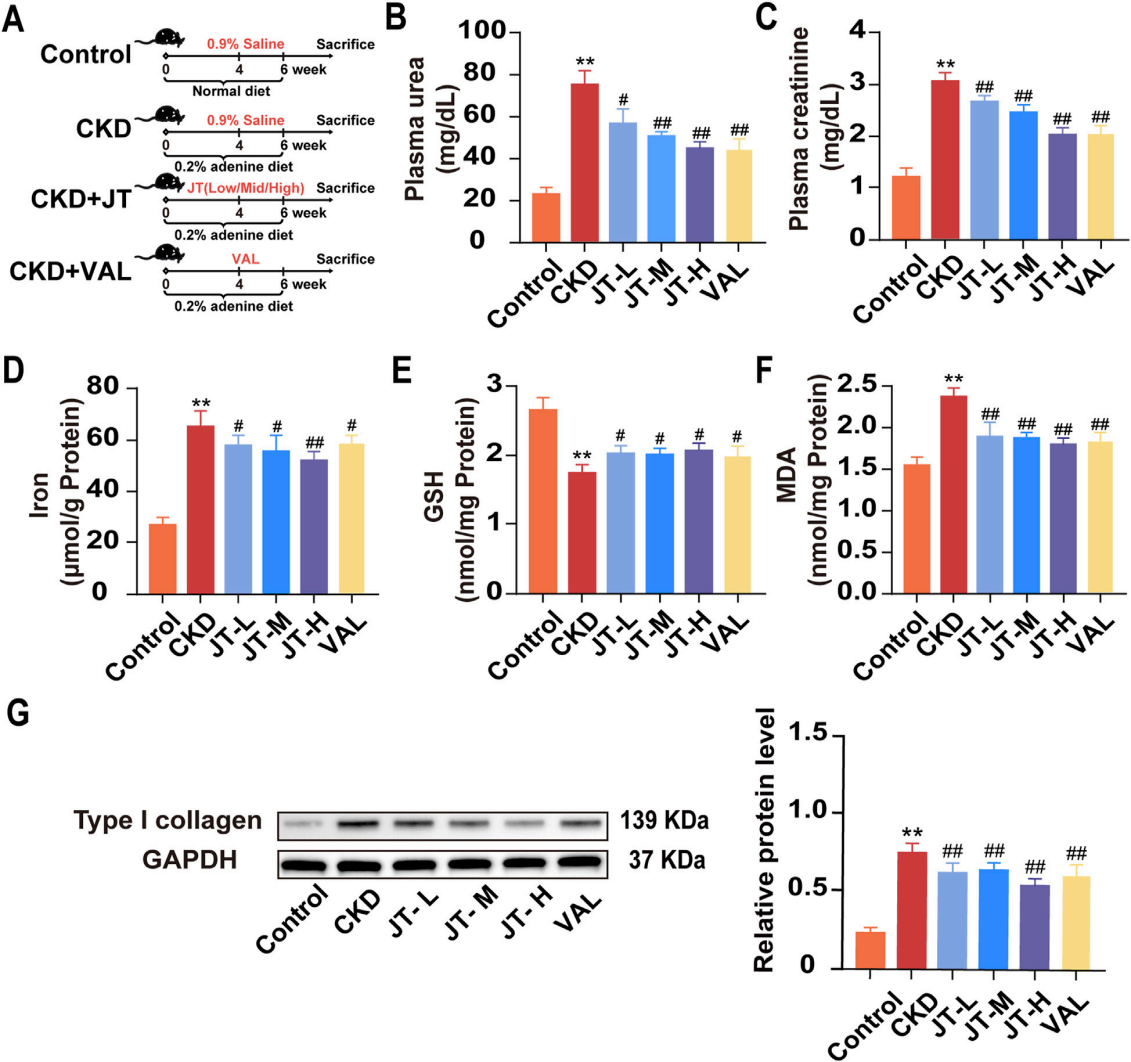
Effects of JT on physicochemical indicators in mice with adenine-induced CKD. **(A)** Schematic diagram of the experiment. **(B)** Plasma urea levels. **(C)** Plasma creatinine levels. **(D)** Total iron content in the kidneys. **(E)** GSH content in the kidneys. **(F)** MDA content in the kidneys. **(G)** Level of type I collagen was evaluated via Western blotting. Densitometric assessment of type I collagen protein levels, with GAPDH used for normalization. Data are presented as the means ± SDs (n = 9). Compared with the control group: *P < 0.05 and **P < 0.01; compared with the CKD group: ^#^ *P* < 0.05 and ^##^ *P* < 0.01.

One microliter each of the mixed control solution described in Section 2.3.2 and the test solution of JT described in Section 2.3.1 was added, and the mixture was injected into the ultrahigh performance liquid chromatograph according to the chromatographic conditions described in Section 2.1. The chromatographic peaks and separation of 5-hydroxymethylfurfural, *R. rosea* glycoside, chlorogenic acid, cinnamic acid, rhubarbic acid, ammonium glycyrrhizinate, rhodopsin, glycyrrhizic acid, rhubarb phenol, and *Aloe barbadensis* rhodopsin were good, as shown in Figure 1.

### 3.2 Establishment of fingerprints of Jingtian particles

The UPLC chromatograms of the 13 batches of samples were obtained by preparing test solutions of the 13 batches of samples according to the method described in Section 2.3.1, injecting the samples according to the chromatographic conditions described in Section 2.3, and then importing them into the software “Fingerprint Evaluation System of Traditional Chinese Medicine Chromatograms (2012 version)” for analysis and evaluation. The similarity of the fingerprint profiles was above 0.9, and the similarity of the profiles of the Jingtian granules from the 13 batches was 0.985, 0.993, 0.992, 0.996, 0.983, 0.989, 0.955, 0.994, 0.978, 0.980, 0.994, 0.995, and 0.995, which indicated that the samples from each batch were highly similar to each other and that the process used to prepare the Jingtian granules from each batch was relatively stable. The granule preparation process was relatively stable, and the quality consistency was good.

### 3.3 Effects of JT on kidney function indicators, lipid peroxidation levels, and iron levels in the kidney tissue of mice with adenine-induced CKD

The experimental flowchart is shown in Figure 1A. The data indicated that mice with adenine-induced CKD had significantly higher serum urea and creatinine concentrations than did the control mice. Following JT therapy, the concentrations of these biochemical markers were reversed in a dose-dependent manner, similar to the effects of valsartan (Figures 1B,C). In contrast to those in the group under supervision, iron (Figure 1D) and MDA (Figure 1F) levels in kidney tissue decreased in a dose-dependent manner after the JT intervention. Conversely, the GSH levels in the kidney tissue showed the opposite trend (Figure 1E). JT treatment was also associated with decreased typeI collagen protein levels (Figure 1G). In summary, these results demonstrate that JT can alleviate fibrosis levels in adenine-induced mice and reduce the expression of MDA and GSH in the kidneys.

### 3.4 Systemic regulation of renal mRNA expression by JT in CKD mice

**FIGURE 2:**
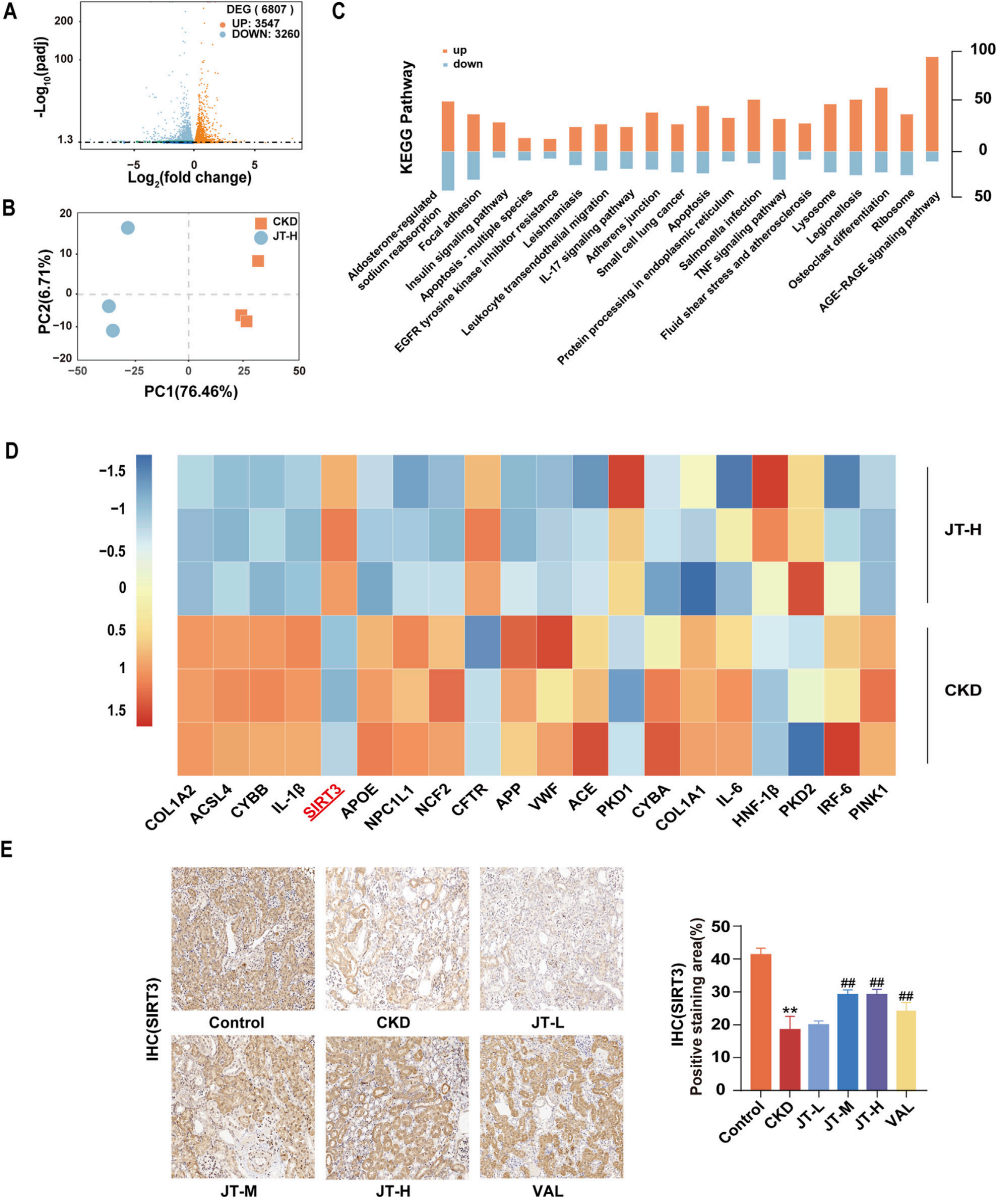
RNA-seq analysis of mouse kidney samples. **(A)** Volcano plot showing upregulated (red dots) and downregulated (blue dots) mRNAs in the comparisons. **(B)** PCA plot displaying three distinct clusters, revealing differences in mRNA expression between the two experimental groups. **(C)** KEGG pathway analysis revealing relevant pathways. **(D)** Heatmap showing the upregulation (red) and downregulation (blue) of specific targets in the comparisons. **(E)** Expression of SIRT3 in mouse kidney tissue (400×, scale bar = 50 μm). Data are presented as the means ± SDs (n = 9). Compared with the control group: *P < 0.05 and **P < 0.01; compared with the CKD group: ^#^ *P* < 0.05 and ^##^ *P* < 0.01.

After confirming the renoprotective effect of JT, RNA-seq analysis was performed to identify mRNAs that were differentially expressed in the kidneys of CKD mice before and after JT therapy to investigate the antifibrotic mechanism of JT. After the RNA-seq data were normalized and filtered, 6807 differentially expressed mRNAs were identified: 3,547 upregulated and 3,260 downregulated (Figure 2A). Principal component analysis (PCA) revealed three distinct clusters, highlighting differences in mRNA expression between the two experimental groups (Figure 2B). The Kyoto Encyclopedia of Genes and Genomes (KEGG) analysis revealed that the mechanism of action of JT might involve the AGE-RAGE signaling pathway, apoptosis, the TNF-α signaling pathway, and the IL-17 and EGFR signaling pathways (Figure 2C). Notably, 20 mRNAs commonly associated with kidney diseases were significantly differentially expressed (Figure 2D). All analyses were based on an FPKM > 1 and absolute log fold change ≥ 1, defining log2 FC > 1 as upregulated and log2 FC < −1 as downregulated. Additionally, the expression of SIRT3 (a longevity gene of recent interest) and acyl-CoA synthetase long-chain family member 4 (ACSL4, a critical protein in ferroptosis) changed significantly. SIRT3 protein expression in kidney tissue was analyzed via immunohistochemistry to explore the “SIRT3-ferroptosis-fibrosis” pathway. The findings revealed a noteworthy increase in SIRT3-positive areas in JT-treated CKD mice (Figure 2E), suggesting that JT treatment affects the ferroptosis pathway.

### 3.5 Effect of JT on the pathological morphology of the kidney tissue in CKD mice

**FIGURE 3:**
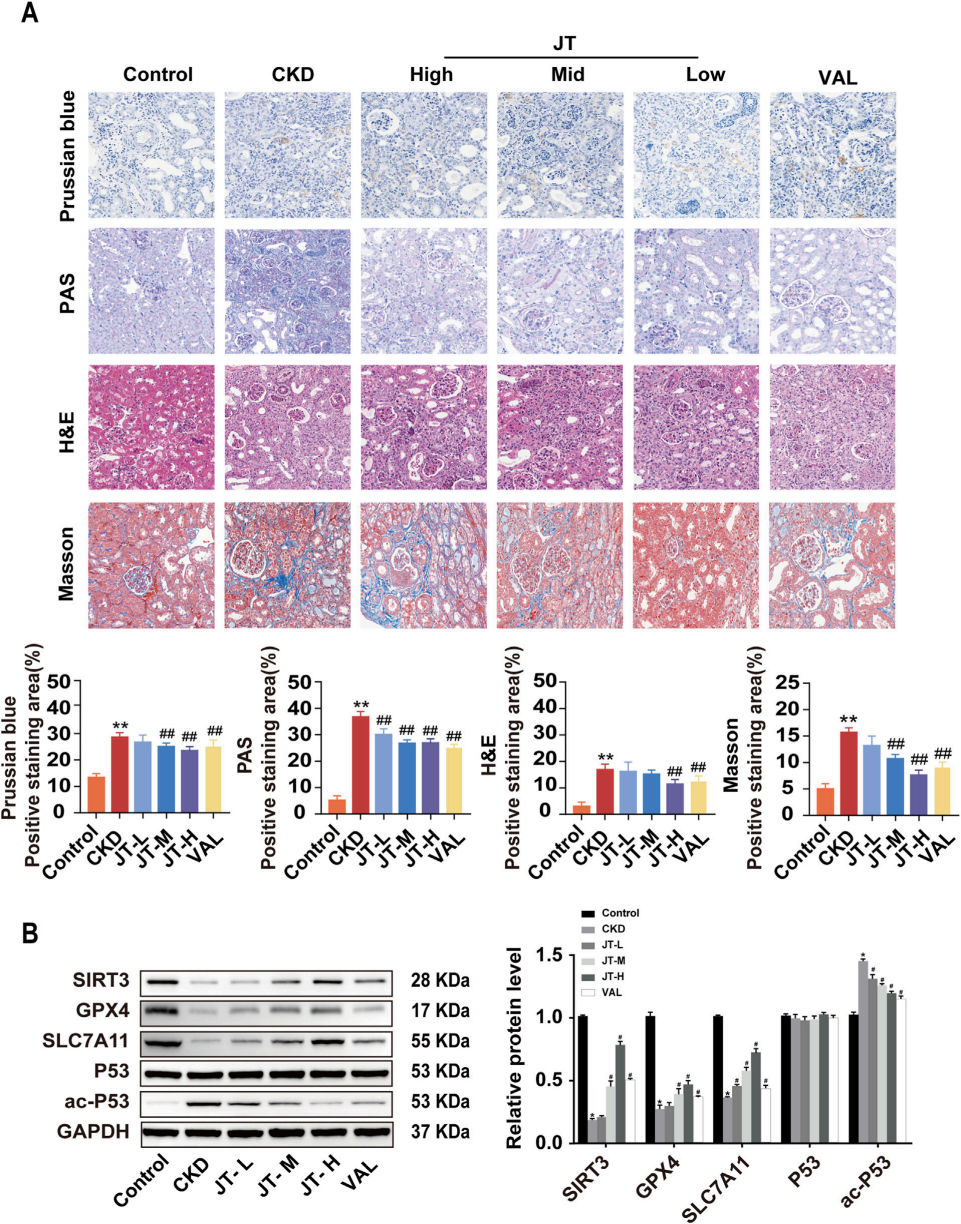
Inhibitory effects of JT on the iron content, inflammatory response, and fibrosis in the kidney tissue of CKD mice. **(A)** Prussian blue staining (400×, scale bar = 50 μm) shows iron deposition in mouse kidney tissues; PAS staining (400×, scale bar = 50 μm) shows inflammation and other lesions in the renal tubules; H&E staining (400×, scale bar = 50 μm) shows pathological changes in the renal interstitium and glomeruli; and Masson’s trichrome staining (400×, scale bar = 50 μm) shows collagen fiber deposition in the renal interstitium and glomeruli. **(B)** Western blot analysis of proteins related to the ferroptosis signaling pathway: SIRT3, GPX4, SLC7A11, P53, and ac-P53,with GAPDH used for normalization. Data are presented as the means ± SDs. Compared with the control group: *P < 0.05 and **P < 0.01; compared with the CKD group: ^#^ *P* < 0.05 and ^##^ *P* < 0.01.

H&E staining revealed that the glomeruli, renal tubules, and renal interstitial structure of the mice in the control group were normal. In the CKD group, the pathological changes included glomerular hypertrophy, renal tubular epithelial cell vacuolization, protein casts in the renal interstitium, and inflammatory cell infiltration. JT treatment reversed these histopathological changes, reducing inflammation and renal tubule atrophy. Prussian blue staining indicated that compared with the control, the JT intervention reduced Fe^2+^ deposition in renal tubules. Masson’s trichrome staining revealed a significant reduction in interstitial fibrosis in the CKD group after treatment. PAS staining revealed a significant disruption of the structural integrity and tubular dilation in CKD mice, effects that were partially reversed by the JT intervention. Western blot analysis revealed that SIRT3 protein expression was significantly decreased in the CKD group compared with the control group, whereas the JT intervention increased SIRT3 protein expression in a dose-dependent manner. GPX4 and SLC7A11 expression decreased in the CKD group but increased after treatment with different doses of JT. No discernible difference in P53 expression was detected across the groups, but the level of the acetylated form of P53 increased significantly in the CKD group and decreased significantly with JT and VAL treatment (Figure 3B). These results confirm the successful establishment of the adenine-induced CKD mouse model and show that JT has the potential to alleviate renal damage through a mechanism possibly related to ferroptosis.

### 3.6 Optimal concentration of JT determined by the CCK-8 assay

**FIGURE 4:**
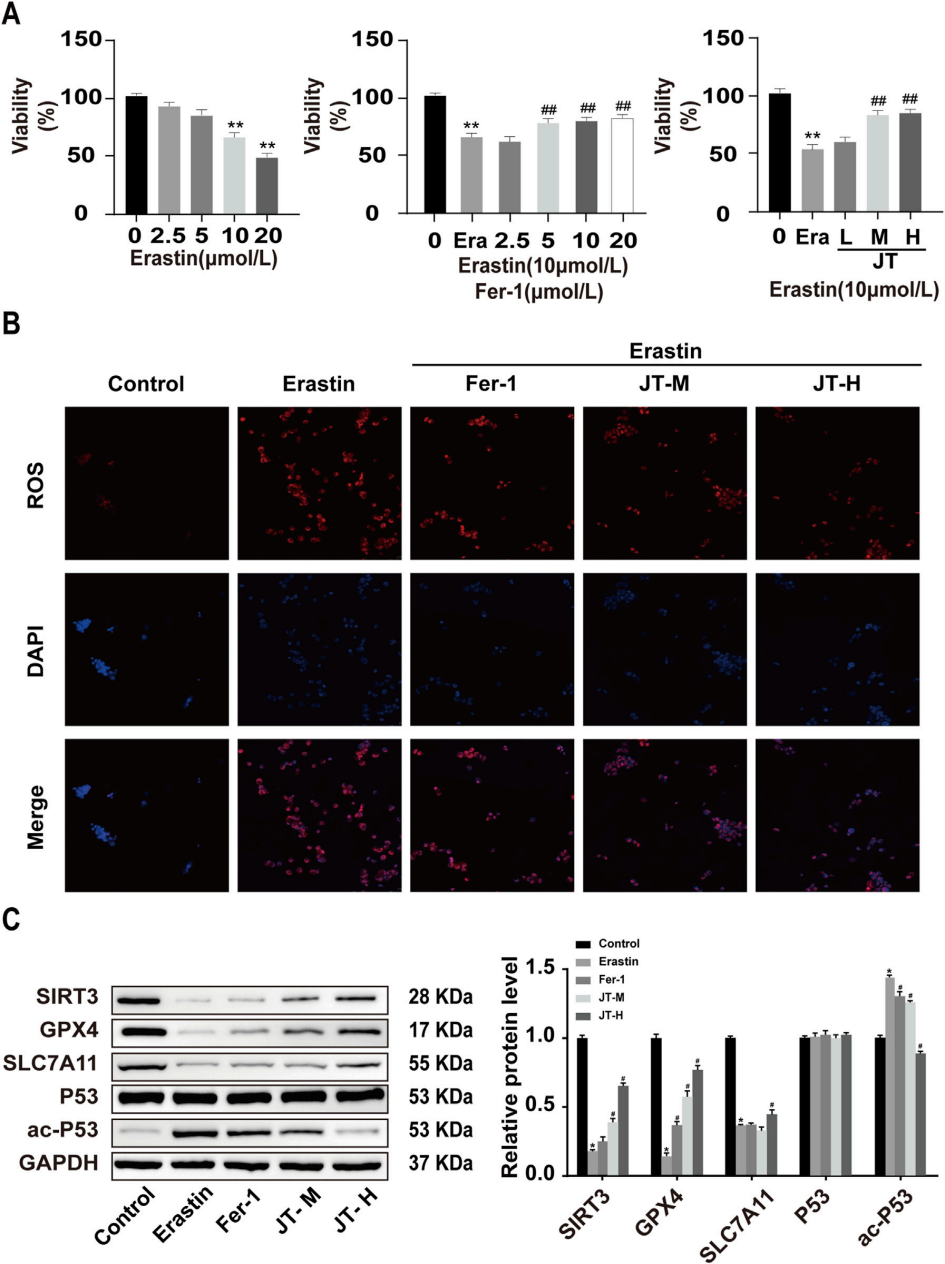
Effects of JT on the activity and ROS levels of erastin-damaged HK-2 cells. **(A)** CCK-8 assay to determine the optimal concentrations of erastin, Fer-1, and JT. Effects of JT on the morphology of erastin-damaged HK-2 cells (scale bar = 50 μm) **(B)** ROS levels in HK-2 cells (400×, scale bar = 50 μm). Data are presented as the means (SEMs). n = 3. Compared with the control group: *P < 0.05 and **P < 0.01; compared with the erastin group: ^#^ *P* < 0.05 and ^##^ *P* < 0.01.**(C)** Western blot analysis of the protein levels of SIRT3, GPX4, SLC7A11, P53, and ac-P53 in HK-2 cells, with GAPDH used for normalization. Data are presented as the means ± SDs. Compared with the control group: *P < 0.05 and **P < 0.01; compared with the erastin group: ^#^ *P* < 0.05 and ^##^ *P* < 0.01.

The results of the CCK-8 assay revealed that 10 μmol/L erastin inhibited HK-2 cell activity after 24 h, following a dose‒response curve. Therefore, 10 μmol/L erastin was used to induce HK-2 cell injury in subsequent experiments. Similarly, based on the results of the cell viability assay, 5 μmol/L Fer-1 was used as the intervention dose. Medium and high doses of JT-containing serum promoted the activity of erastin-treated HK-2 cells; therefore, medium and high doses of JT were chosen for the cell-based experiments (Figure 4A).

### 3.7 Effects of erastin, Fer-1, and JT on ROS levels in HK-2 cells

The ROS staining results revealed low ROS levels in the control group. Compared with those in control cells, the ROS fluorescence signals were more intense in erastin-stimulated HK-2 cells. The ROS signal intensity in the erastin+Fer-1 group was weaker than that in the erastin group. Compared with those in HK-2 cells in the erastin group, the ROS fluorescence signals in the medium- and high-dose JT groups were less pronounced (Figure 4B).

### 3.8 Effect of JT on the Sirt3/P53/GPX4 signaling pathway in erastin-damaged HK-2 cells

The results of the Western blot analysis revealed a considerably higher level of the ac-P53 protein in the erastin group than in the control group, whereas it was lower in the Fer-1, JT-M, and JT-H groups. The erastin group presented decreases in Sirt3, GPX4, and SLC7A11 protein expression, whereas the Fer-1, JT-M, and JT-H groups presented substantial increases in the expression of these proteins. P53 protein expression did not change significantly in any of the groups (Figure 4C).

### 3.9 JT does not significantly alter biochemical indicators in SIRT3^−/−^ mice with CKD

**FIGURE 5:**
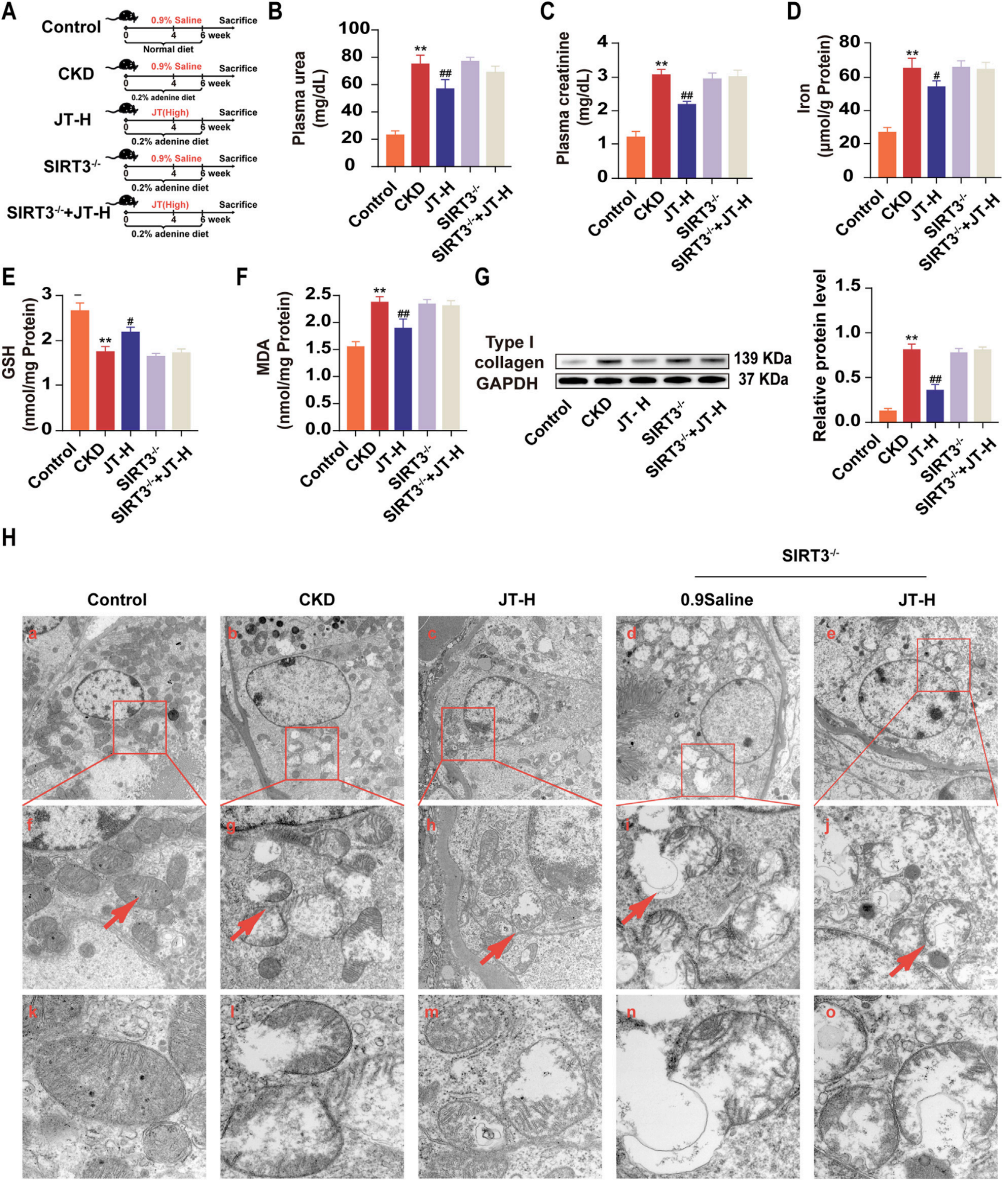
Effects of JT on physicochemical indicators in adenine-fed SIRT3^−/−^ mice. **(A)** Schematic diagram of the experiment. **(B)** Plasma urea levels. **(C)** Plasma creatinine levels. **(D)** Total iron content in the kidneys. **(E)** GSH content in the kidneys. **(F)** MDA content in the kidneys. **(G)** Level of type I collagen was evaluated via Western blotting. Densitometric assessment of type I collagen protein levels, with GAPDH used for normalization. **(H)** Mitochondria observation by TEM analysis, Images f-j are representative areas indicated by the red wireframes in a-e. Images k-o show representative mitochondria indicated by red arrows in f-j. a-c, scale bar = 5 μm; d-f, scale bar = 2 μm; g-i, scale bar = 500 nm. Data are presented as the means ± SDs. Compared with the control group: *P < 0.05 and **P < 0.01; compared with the CKD group: ^#^ *P* < 0.05 and ^##^ *P* < 0.01.

**FIGURE 6:**
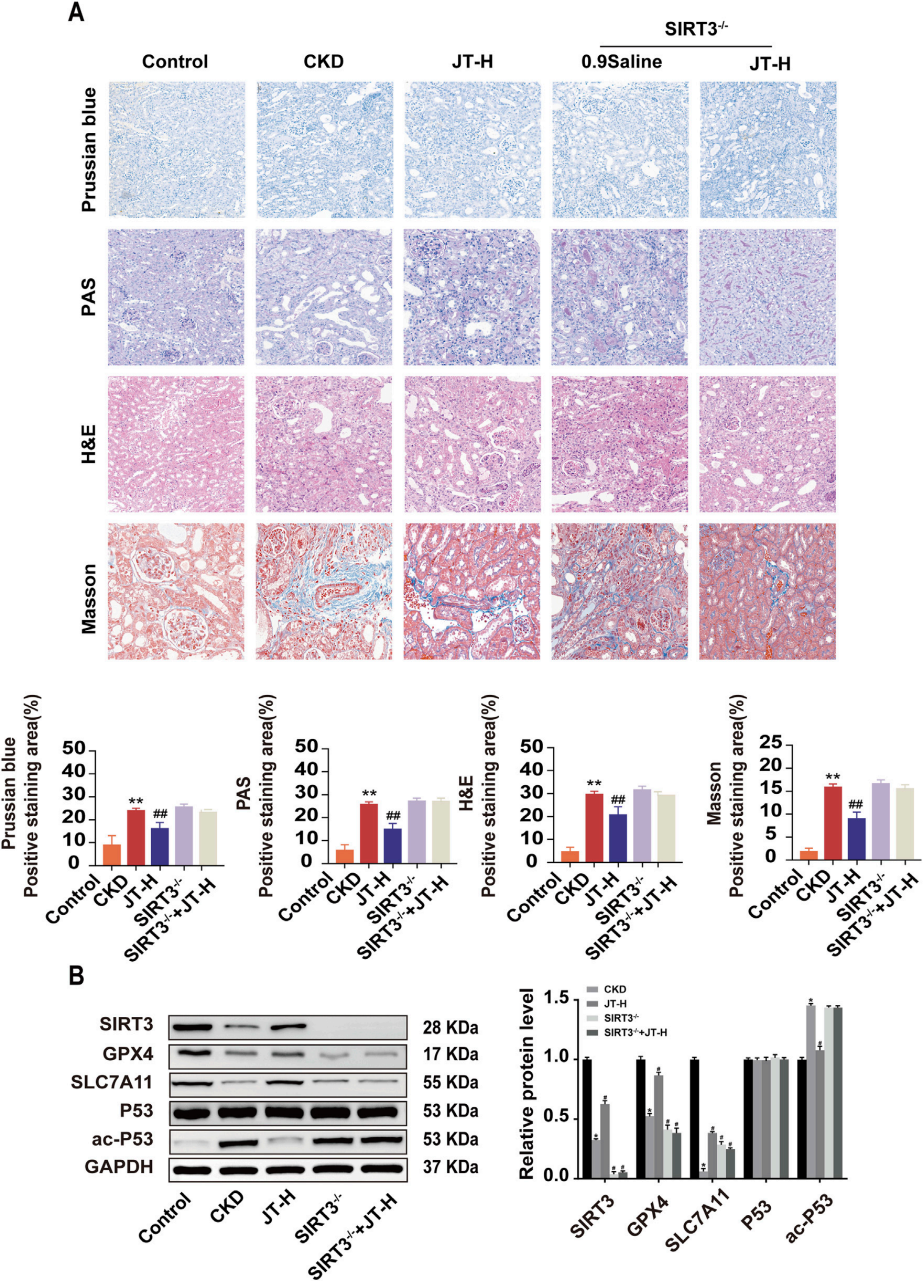
Inhibitory effects of JT on the iron content, the inflammatory response, and fibrosis in the kidney tissues of SIRT3^−/−^ mice. **(A)** H&E staining (400×, scale bar = 50 μm) shows pathological changes in the renal interstitium and glomeruli; Masson’s trichrome staining (400×, scale bar = 50 μm) shows collagen fiber deposition in the renal interstitium and glomeruli; PAS staining (400×, scale bar = 50 μm) shows inflammation and other lesions in the renal tubules; and Prussian blue staining (400×, scale bar = 50 μm) shows iron deposition in the mouse kidney tissue. **(B)** Western blot analysis of proteins related to the ferroptosis signaling pathway: SIRT3, GPX4, SLC7A11, P53, and ac-P53,with GAPDH used for normalization. Data are presented as the means ± SDs. Compared with the control group: *P < 0.05 and **P < 0.01; compared with the CKD group: ^#^ *P* < 0.05 and ^##^ *P* < 0.01.

Experiments were conducted with control mice fed a normal diet, adenine diet, or adenine diet+JT-H and with SIRT3^−/−^ mice fed an adenine diet or adenine diet+JT-H to evaluate the role of SIRT3 in the protective effect of JT on CKD (Figure 5A). Significant renal fibrosis progression was observed after 6 weeks of adenine diet feeding. As shown in Figure 6, most of the deteriorating physicochemical parameters, including the serum urea, creatinine, GSH, MDA, and iron levels, in the CKD group did not improve in the SIRT3^−/−^ and SIRT3^−/−^+JT-H groups (Figures 5B–F). Under JT treatment, the protein expression level of type I collagen was reduced. In contrast, after SIRT3 knockout, its expression level did not show significant changes compared with the CKD group (Figure 5G). We subsequently employed TEM to assess the ultrastructural changes in renal tubular cells following injury or necrosis. The TEM results revealed that in the CKD model group, the mitochondria in proximal renal tubular cells exhibited significant morphological alterations, characterized by decreased volume and reduced or absent mitochondrial cristae. Following JT treatment, these mitochondrial ultrastructural defects were reversed. However, this reversal was not observed in SIRT3 knockout mice (Figure 5H). In conclusion, these findings demonstrate that SIRT3 plays a crucial role in mediating the protective effects of JT on renal fibrosis and mitochondrial integrity in CKD. The absence of SIRT3 abrogates the beneficial effects of JT on renal function, oxidative stress markers, and mitochondrial ultrastructure, highlighting its essential contribution to the therapeutic potential of JT in CKD.

### 3.10 JT does not significantly normalize the renal structure and related protein expression in SIRT3^−/−^ mice with CKD

Compared with wild-type mice, SIRT3 knockout mice did not exhibit any protective effects on the renal pathological structure, fibrosis, or iron deposition (Figure 6A). Furthermore, a protein blotting analysis was performed to assess the expression of related signaling proteins in the kidneys to study the impact of JT on renal fibrosis signaling pathways. Under CKD conditions, GPX4 and SLC7A11 expression decreased; however, the expression of these proteins did not increase in the absence of SIRT3. Conversely, P53 levels remained unchanged in all groups, although ac-P53 levels were significantly increased in CKD mice (Figure 6B). These results suggest that JT does not significantly alleviate CKD progression in SIRT3^−/−^ mice.

## 4 Discussion

Renal fibrosis encompasses interstitial fibrosis, glomerulosclerosis, and arteriosclerosis (Zoccali et al., 2017). Research indicates that the renal interstitium is more crucial than the glomeruli in maintaining normal kidney function and in determining the CKD prognosis. The structure and function of the renal interstitium, which is situated between the tubules and blood vessels, are closely related to the tubules. Interstitial damage frequently accompanies tubular injury. Tubulointerstitial fibrosis is both a hallmark and a final pathological outcome of many CKD cases. Preventing and treating tubulointerstitial fibrosis can delay the progression of CKD (Obrador and Levin, 2019). Unfortunately, our understanding of renal fibrosis pathobiology is limited, and clinical strategies are scarce. Therefore, establishing animal models to study renal interstitial fibrosis mechanisms is crucial. This research requires rethinking the fundamental mechanisms driving renal fibrosis and identifying new therapeutic targets and methods.

In this study, an adenine-containing diet was fed to animals to induce CKD and better simulate the structural and functional changes in humans with CKD. The primary mechanism of adenine-induced CKD in mice involves the formation of insoluble 2,8-dihydroxyadenine, which crystallizes in the renal tubules, leading to kidney injury (Barton et al., 2022). Compared with other models, adenine-induced CKD involves extensive renal and cardiovascular dysfunction, including increased inflammation, fibrosis, and oxidative marker expression in the kidneys, effects that are consistent with the chronic nature of CKD. The adenine-induced model has helped researchers better understand the pathophysiological mechanisms of CKD and test new treatments (Mohammed-Ali et al., 2015). As shown in Figures 1B,C, mice fed adenine had elevated plasma creatinine and blood urea nitrogen levels, with no differences in dietary intake between the groups. This finding indicates that the differences in physical and chemical factors and morphology between groups were not due to differences in adenine intake.

TCM has achieved significant progress in treating renal fibrosis. By regulating various signaling pathways, TCM has shown good efficacy. For example, we reported that the Bupi Yishen formula (BYF) improves renal function and reduces fibrosis symptoms (Liu et al., 2022). Similarly, the Wen Shen Jian Pi Hua Tan Tang (WSHT) formula has shown protective effects on preventing early obesity-related glomerulopathy (ORG) and its potential mechanisms have been identified. In a high-fat diet-induced obese rat model, WSHT significantly improved obesity-induced renal pathological changes, including fat deposition and glomerular hypertrophy (Song et al., 2023). Numerous clinical and animal studies have validated the efficacy of TCM, which exerts enhanced therapeutic effects through multitarget and multipathway comprehensive treatment strategies (Tang et al., 2019).

This study systematically explored the potential of JT in treating CKD, providing multifaceted evidence of its efficacy. First, *in vivo* experiments revealed that JT treatment significantly reduced the serum urea and creatinine levels in adenine-induced CKD model mice, indicating improved renal function. Additionally, JT treatment decreased iron and malondialdehyde (MDA) levels in renal tissues while increasing glutathione (GSH) levels, indicating significant antioxidant effects. MDA is a primary indicator of oxidative stress and a significant byproduct of lipid peroxidation (Estébanez et al., 2022). During ferroptosis, MDA levels increase significantly, reflecting the extent of lipid peroxidation (Jelic et al., 2021). Studies have shown that elevated MDA levels are closely linked to ferroptosis. GSH is the main intracellular antioxidant that scavenges free radicals and peroxides, protecting cells from oxidative damage (Qiu et al., 2024). During ferroptosis, GSH functions via GPX4. GPX4 uses GSH to reduce lipid peroxides to nontoxic lipid alcohols, preventing excess lipid peroxidation. Thus, GSH depletion reduces GPX4 activity, promoting ferroptosis. The histopathological examination confirmed that JT normalized renal tissue morphology, reducing tubular atrophy, inflammatory cell infiltration, and interstitial fibrosis (Li et al., 2023).

Iron-dependent lipid peroxidation damages cell membranes during ferroptosis, a type of programmed cell death; it involves the iron-catalyzed peroxidation of polyunsaturated fatty acids (PUFAs), which produce lipid peroxidation products such as MDA, causing cell damage and death. GPX4 prevents ferroptosis by reducing lipid peroxides (Ma et al., 2022). Renal fibrosis refers to the destruction of the renal parenchyma and accumulation of fibrous connective tissue, a common pathological pathway in chronic and progressive kidney diseases, and histologically manifests as glomerulosclerosis, tubulointerstitial fibrosis, and renal vascular remodeling (Ye et al., 2022). Under the influence of various inflammatory mediators and cytokines, fibroblasts differentiate into myofibroblasts, which proliferate excessively and overexpress alpha-smooth muscle actin, collagen fibers, fibronectin, and matrix metalloproteinases (Feng et al., 2022). Previous studies have shown that the progression of renal fibrosis involves programmed cell death, including necrosis, autophagy, and apoptosis. For example, studies have shown that salidroside improves renal function and reduces collagen fiber deposition by regulating iron metabolism and inhibiting ferroptosis, significantly alleviating renal fibrosis in SAMP8 mice (Yang et al., 2022). Recent studies on ferroptosis-related metabolic pathways and their roles in kidney diseases have revealed the significant role of ferroptosis in renal fibrosis pathology (Portilla, 2014).

Studies revealed the molecular mechanism of action of JT. RNA-seq and Western blot analyses revealed that JT regulated key proteins in the SIRT3/P53/GPX4 signaling pathway. Specifically, JT treatment increased the expression of SIRT3, GPX4, and SLC7A11 while reducing the level of acetylated P53, indicating that JT plays a role in regulating the SIRT3/P53/GPX4 pathway. SIRT3 knockout mice were used in experiments to identify the key role of SIRT3 in JT treatment. The results revealed that without SIRT3, the protective effects of JT were significantly weakened, confirming the importance of SIRT3 in JT treatment. Cellular experiments supported these findings. By reducing ROS levels, JT markedly upregulated SIRT3, GPX4, and SLC7A11 expression in erastin-treated HK-2 cells while decreasing acetylated P53 levels, further confirming that the nephroprotective mechanism of action of JT is mediated by the SIRT3/P53/GPX4 axis. These findings provide a new perspective for the application of TCM in CKD treatment and highlight the key role of SIRT3 in therapeutic strategies.

Mitochondrial Sirtuin 3 (SIRT3) is part of the conserved NAD+-dependent deacetylase family; it regulates the mitochondrial respiratory chain, ATP generation, and fatty acid β-oxidation, and possesses antioxidant activity (Huang et al., 2022). The kidneys are among the organs that consume the most ATP, and mitochondrial alterations are a hallmark of kidney diseases. In our study, we found that SIRT3 absence does not directly worsen renal fibrosis but significantly diminishes the therapeutic effect of JT. In the adenine-induced CKD model, SIRT3 knockout did not exacerbate fibrosis compared to the model group, indicating that SIRT3 absence alone does not directly worsen fibrosis. However, JT showed a clear anti-fibrotic effect, especially at high doses. Importantly, this effect was absent in SIRT3 knockout mice treated with high-dose JT, suggesting that JT’s anti-fibrotic action relies heavily on SIRT3 activation. The lack of improvement in renal fibrosis with high-dose JT in SIRT3^−/−^ mice further confirms SIRT3’s central role in JT’s therapeutic mechanism. Mitochondria, the “powerhouses of the cell”, provide over 90% of cellular energy; they also participate in cell signaling, proliferation, and death. Recent research highlights the importance of SIRT3 as a lysine deacetylase and regulator of mitochondrial stress responses (Perico et al., 2016). These findings provide new therapeutic targets for improving end-organ damage. The tumor suppressor protein P53 (TP53) is crucial for antitumor function because it regulates cell death and the cell cycle. P53 is closely related to ferroptosis, particularly in promoting ferritin degradation (Wang et al., 2019). This process affects the GPX4-regulated pathway, ultimately leading to ferroptosis. Mechanistically, P53 promotes USP7 nuclear translocation. This process reduces H2B monoubiquitination at lysine 120 (H2Bub1) in the SLC7A11 gene regulatory region, leading to SLC7A11 inactivation (Mao et al., 2019). ROS significantly oxidize lipids, proteins, and DNA; damage tubules and the vascular endothelium; and promote the secretion of profibrotic factors. ROS have the potential to prevent fibrosis by promoting fibroblast activation and endothelial‒mesenchymal transition (EndMT) (Morry et al., 2017). HK-2 cells secrete profibrotic factors during ferroptosis, promoting fibroblast proliferation. Increased ROS levels in podocytes directly damage podocytes and stimulate the EndMT (Chang et al., 2019). Through increased lipid ROS production and membrane rupture, ferroptosis leads to the release of DAMP signals, triggering inflammation and immune responses, making it a therapeutic target for renal fibrosis. Erastin and TGF-β induce ferroptosis and promote fibrosis, and ureteral obstruction induces ferroptosis, with HK-2 cells secreting profibrotic factors, further promoting fibroblast proliferation and activation (Yu et al., 2022). Since ROS are involved in the proapoptotic function of P53, their production is now considered the basis of P53-induced cell death. Moreover, P53-induced apoptosis depends on ROS production and the release of proapoptotic factors in damaged cells, effects that were strongly confirmed in this study.

Previous studies have confirmed that P53 deacetylation alleviates renal fibrosis induced by calcium oxalate crystals by inhibiting ferroptosis, suggesting that SIRT1-mediated P53 deacetylation is a potential target for treating renal fibrosis (Mao et al., 2019). Combined with the RNA-seq results, these findings suggest that the key mechanism of action of JT in inhibiting renal fibrosis may involve the activation of SIRT3/ac-P53 signaling. The results of these experiments indicated that JT activated SIRT3/ac-P53 and inhibited ferroptosis both *in vivo* and *in vitro*. We also generated SIRT3 knockout mice to verify the renoprotective mechanism of JT. The results showed that the therapeutic effect of JT was less effective in SIRT3 knockout mice than in wild-type mice, indicating that JT may inhibit fibrosis progression by inducing SIRT3 to deacetylate P53.

## 5 Conclusion

**FIGURE 7:**
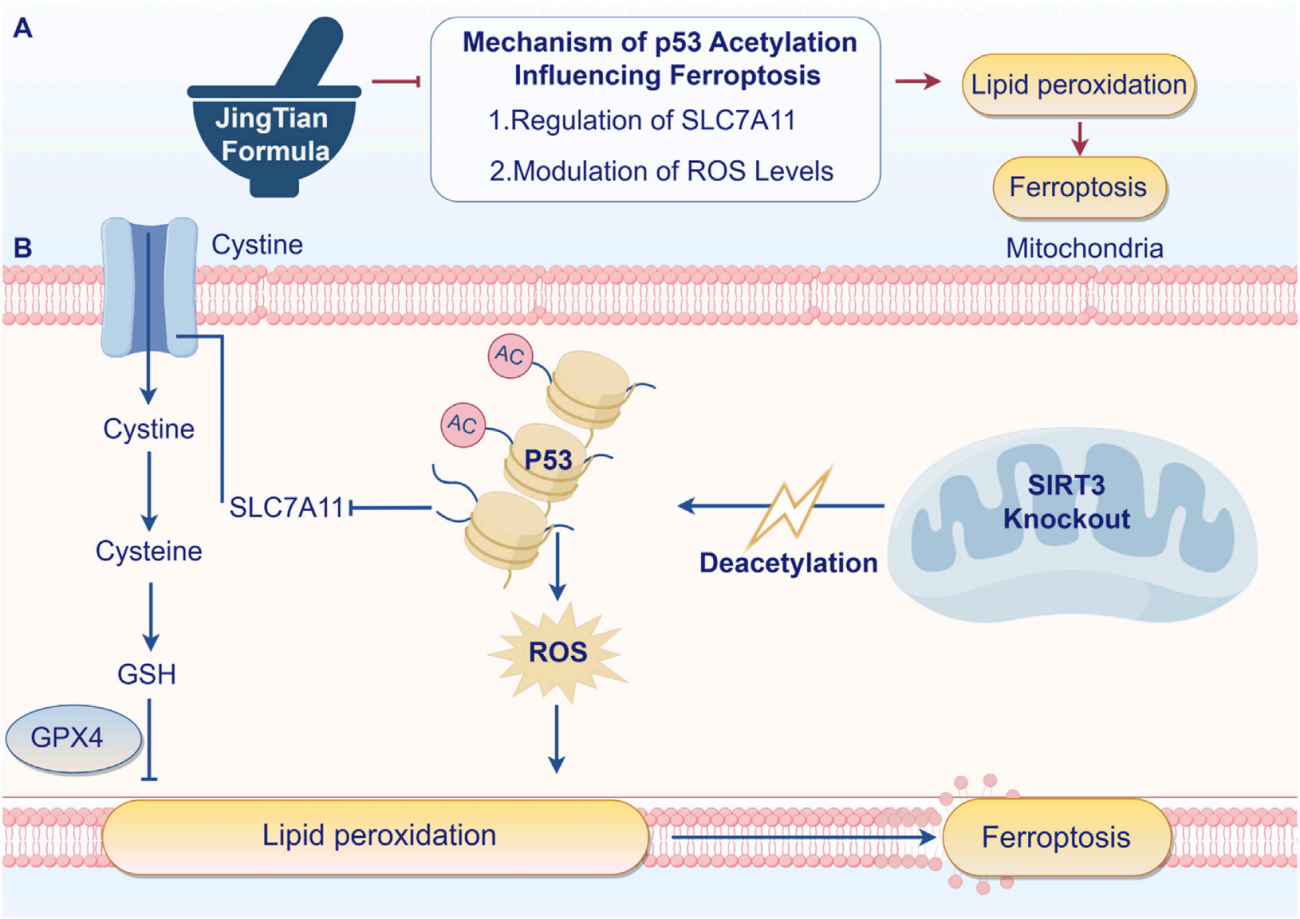
JT inhibits renal fibrosis through SIRT3 by participating in P53 deacetylation and blocking ferroptosis - induced lipid peroxidation. Arrows indicate increased changes after JT treatment. Turnstiles indicate decreased changes after JT treatment (By Figdraw).

In conclusion, our study provides compelling evidence that JT can mitigate renal fibrosis and improve renal function in a CKD model through mechanisms involving the SIRT3/P53 signaling pathway and the inhibition of ferroptosis. These findings offer a new perspective on the potential of TCM formulations in the management of CKD and highlight the importance of molecular pathways that involve SIRT3 in therapeutic strategies. Further research and clinical trials are essential to validate these findings and potentially translate them into effective treatment strategies for CKD patients (Figure 7).

## Data Availability

The data presented in the study are deposited in the NCBI (BioProject) repository, accession number PRJNA1229124.

## Abbreviations

CKD, chronic kidney disease; JT, Jingtian granule; RNA-seq, RNA sequencing; PCA, principal component analysis; KEGG, Kyoto Encyclopedia of Genes and Genomes; FPKM, fragments per kilobase of exon per million mapped fragments; SIRT3, sirtuin 3; ACSL4, acyl-CoA synthetase long-chain family member 4; GPX4, glutathione peroxidase 4; SLC7A11, solute carrier family 7 member 11; P53, tumor protein 53; TCM, traditional Chinese medicine; ROS, reactive oxygen species.
