# Supplementary material for: Jingtian granule alleviates adenine-induced renal fibrosis in mice through SIRT3-Mediated deacetylation of P53
Source: Front Pharmacol. 2025 Mar 12;16:1526414. doi: 10.3389/fphar.2025.1526414 (PMC11936886; doi:10.3389/fphar.2025.1526414)
Supplement: Supplementary file 1 [file DataSheet2.docx]

Supplementary Material

# Supplementary Data

- 1. Creatinine and Urea Analysis

Creatinine and urea levels were measured using colorimetric assay kits from Elabscience Biotechnology Co., Ltd. (Cat. No.: E-BC-K188-M; E-BC-K183-M) according to the instructions.

- 1. Iron, GSH, and MDA Activity Assays

Mouse kidney tissues were cut into small pieces, ground into powder under liquid nitrogen, and homogenized in cold physiological saline. The homogenate was centrifuged at 12,000 rpm for 20 minutes at 4°C, after which the supernatant was retained. The iron content (Cat. No. E-BC-K139-S), GSH content (Cat. No. E-BC-K030-M), and MDA content (Cat. No. E-EL-0060) were measured using kits according to the manufacturer's instructions (Elabscience).

- 1. Hematoxylin and Eosin (H&E) Staining

Fresh tissues were fixed at room temperature with 4% formaldehyde for 30 minutes (Servicebio, Cat. No. G1101-3ML), dehydrated with an ethanol gradient series, embedded in paraffin, and stored at -20°C. Sections (6 µm thick) were deparaffinized with xylene at room temperature, stained with H&E (Servicebio, Cat. No. G1004-500ML), washed with xylene, and sealed with neutral resin.

- 1. Periodic Acid-Schiff (PAS) Staining

PAS staining was performed using a kit from Solarbio (Cat. No. G1281). Paraffin sections were deparaffinized, rinsed in distilled water for 5 minutes, stained with 10 g/L periodic acid for 20 minutes, and rinsed thoroughly with water. The sections were then stained with Schiff reagent for 60 minutes, washed three times with sulfurous acid, and rinsed thoroughly with water. The sections were then washed with tap water, differentiated with hydrochloric acid solution, and blued with ammonia water. Finally, the sections were washed, sealed, and photographed.

- 1. Masson's Trichrome Staining

Masson's trichrome staining was performed using a kit from Solarbio (Cat. No. G1340). Sections were rinsed in double-distilled water for 5 minutes, stained with hematoxylin for 5–10 minutes, and rinsed thoroughly with water. The sections were stained with Masson's light green acid fuchsin solution for 6‒10 minutes, rinsed with 2% cold acetic acid solution for 5 seconds, differentiated with 1% phosphomolybdic acid solution for 3‒5 minutes, directly stained with aniline blue for 5 minutes, and rinsed with 0.2% cold acetic acid solution for a few seconds. The sections were washed, sealed, and photographed.

- 1. Prussian Blue Staining

Prussian blue staining for iron in kidney tissue was performed using a kit from Servicebio (Cat. No. G1029-100ML).

- 1. RNA Sequencing and Data Analysis

Total RNA was extracted from whole kidney tissue using TRIzol reagent (Invitrogen). The RNA content was assessed via electrophoresis and quantified using an RNA 6000 Nano LabChip kit and an Agilent 2100 bioanalyzer (Agilent). Ribosomal RNA was removed using an Epicenter Ribo-Zero Gold Kit (Illumina, San Diego), and 10 µg of total RNA was used to construct RNA-seq libraries for each sample. Residual RNA fragments were cut with divalent cations at high temperature. All the cut RNA fragments were reverse transcribed to construct complementary DNA (cDNA) libraries using an mRNA-seq sample preparation kit (Illumina). The quality of the RNA libraries was assessed using an Agilent 2100 bioanalyzer, and sequencing was performed on the Illumina HiSeq 4000 platform (LC Biotech, Hangzhou, China) with 300 base pair paired-end runs. Adaptor contamination, low-quality bases, and unidentified bases were removed from the raw FASTQ files using Cutadapt (v1.10). Filtered data were mapped to the mouse genome using Bowtie 2 (v2.2.9) and TopHat 2 (v2.0). StringTie (v1.3) and gffcompare were used to assemble mapped reads for each sample using a reference genome method. Transcripts with coding potential were predicted using three tools: the Coding-Non-Coding Index (CNCI) [v2.0], the Coding Potential Calculator (CPC) [v0.9], and Pfam [v1.3]. Transcripts with CPC scores < -1 and CNCI scores < 0 were filtered out. The remaining transcripts were identified as lncRNAs. Perl scripts reassembled all the assembled data from the mouse samples to construct a comprehensive transcriptome. The final transcriptome was obtained, and the abundance of lncRNAs and mRNAs was analyzed using StringTie (v1.3) and quantified as fragments per kilobase of exon per million mapped fragments (FPKM). In all pairwise comparisons, the statistical significance threshold for expressed genes was defined as a fold change ≥1 and FPKM > 1: CKD group vs. JT-H group.

# Supplementary Figures and Tables

## Supplementary Figures


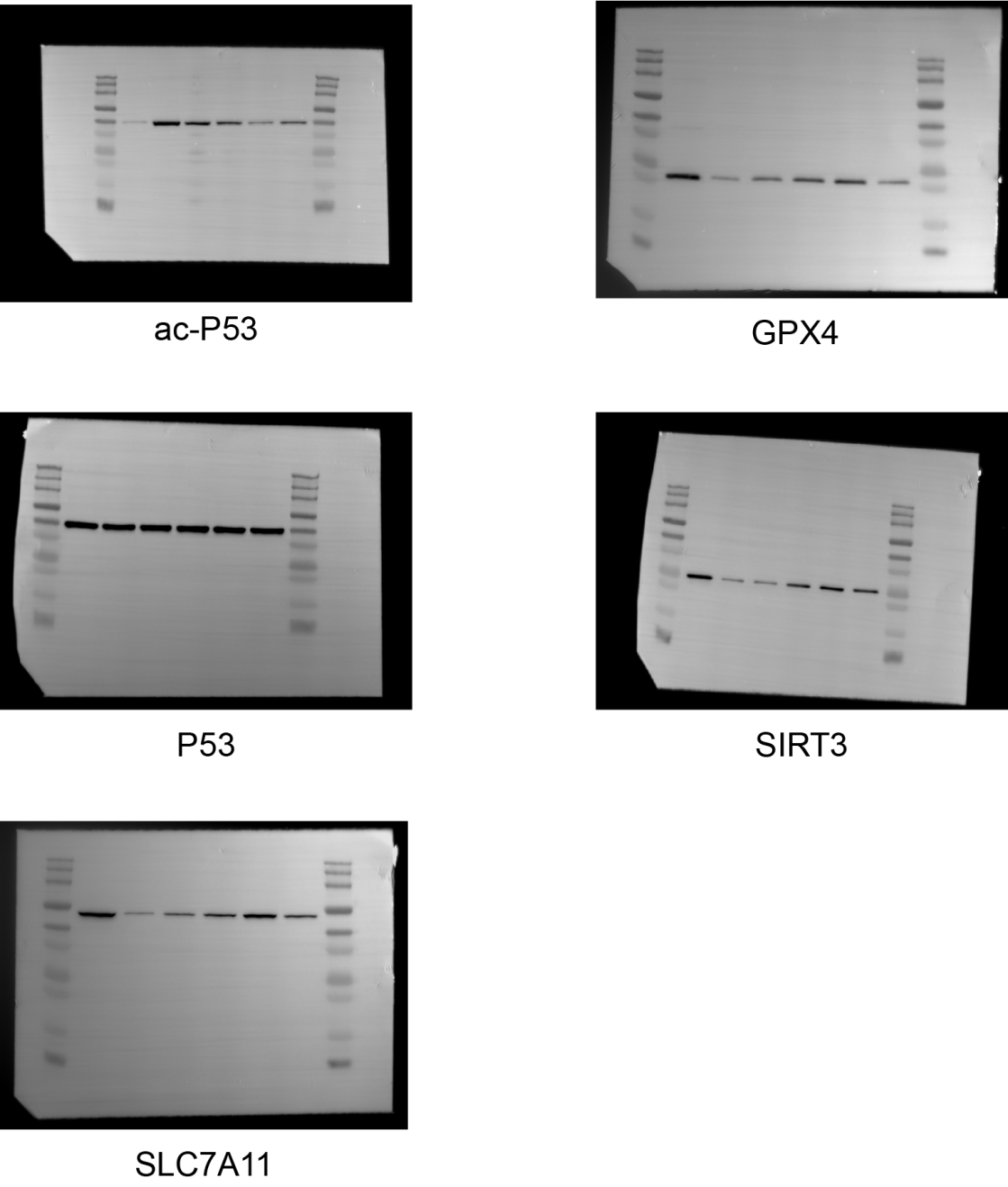


**Supplementary Figure 3.** Uncropped images of original Western blots.


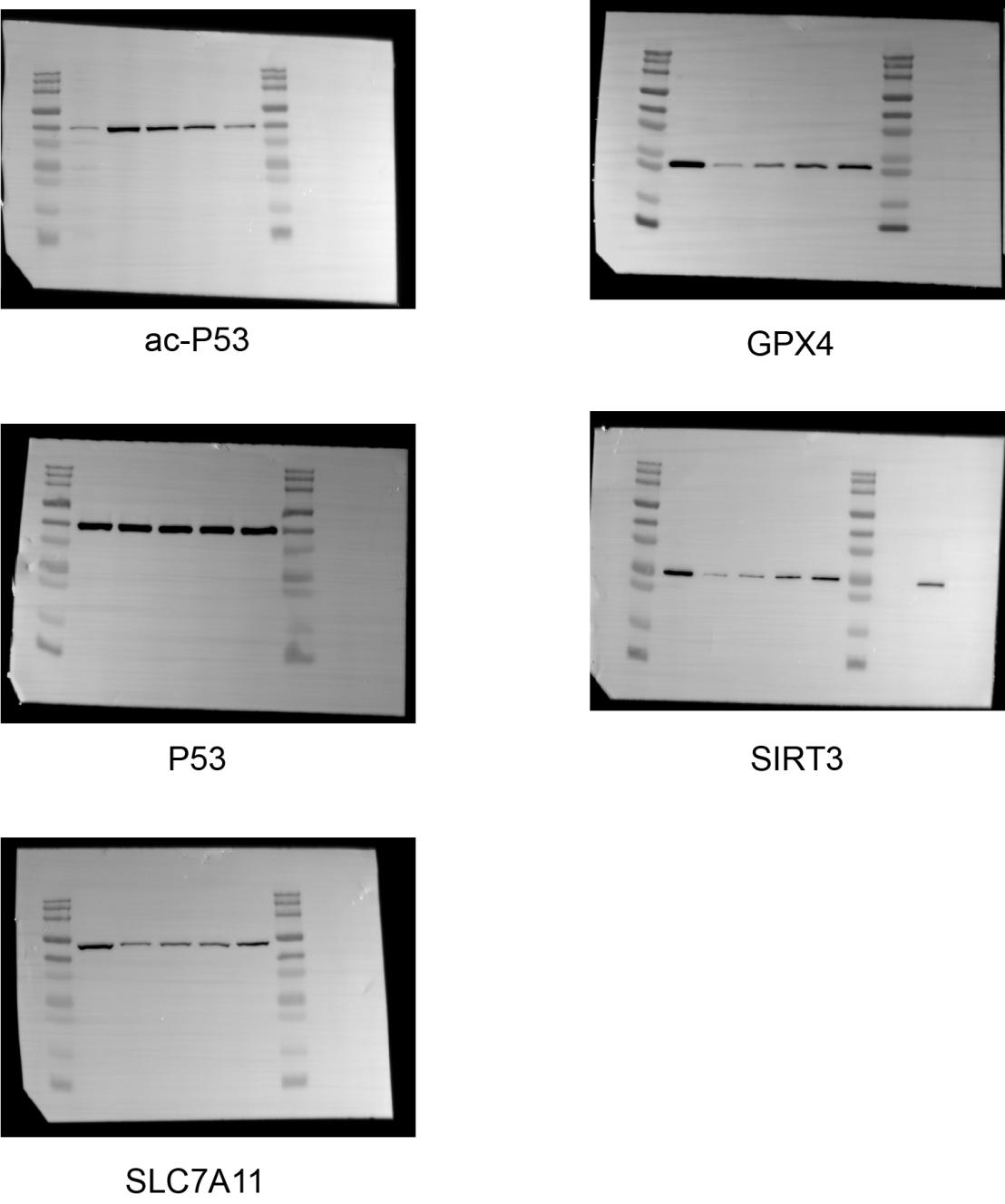


**Supplementary Figure 4.** Uncropped images of original Western blots.


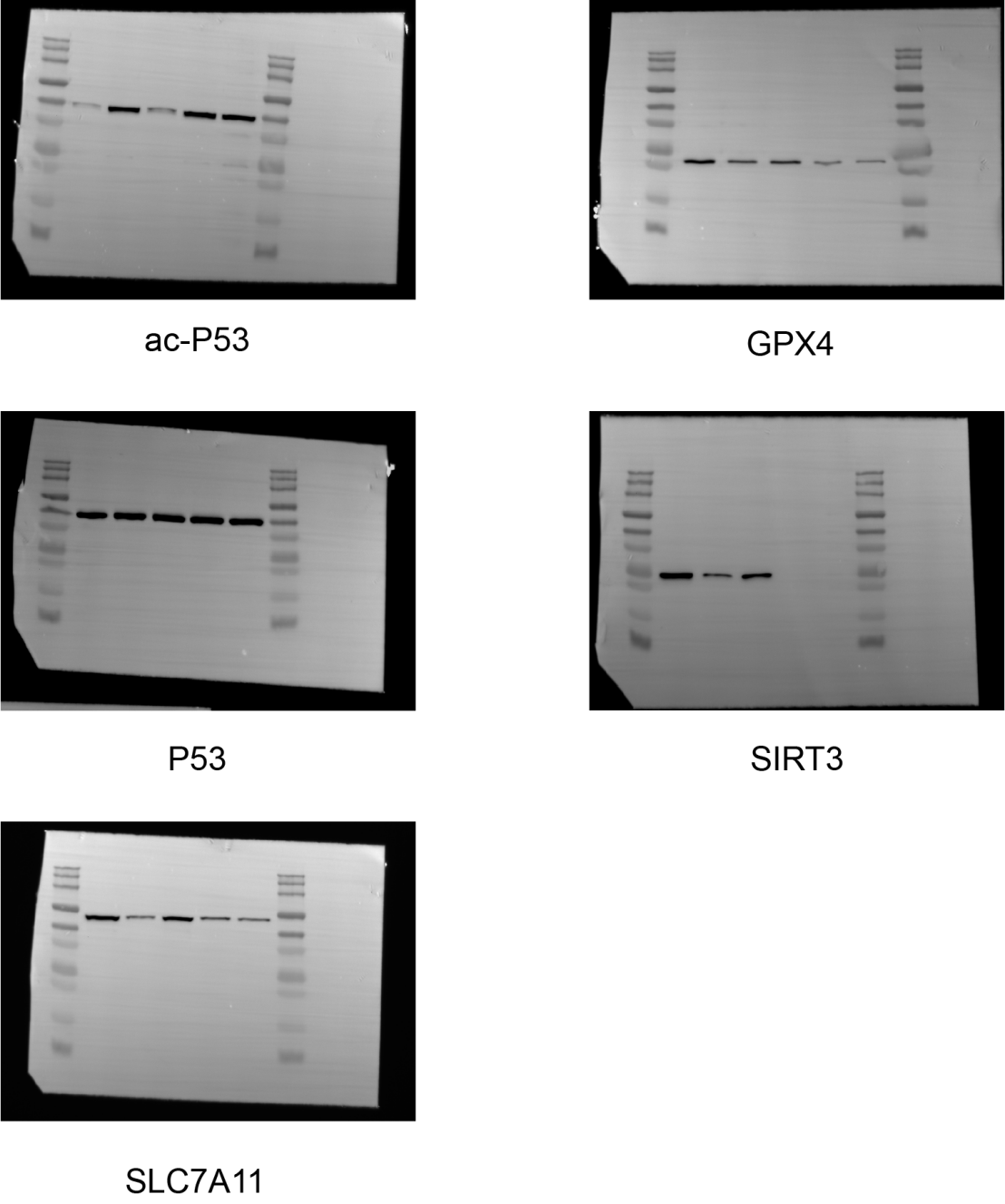


**Supplementary Figure 6.** Uncropped images of original Western blots.
