# Supplementary material for: Jingtian granule alleviates adenine-induced renal fibrosis in mice through SIRT3-Mediated deacetylation of P53
Source: Front Pharmacol. 2025 Mar 12;16:1526414. doi: 10.3389/fphar.2025.1526414 (PMC11936886; doi:10.3389/fphar.2025.1526414)
Supplement: Supplementary file 2 [file DataSheet1.docx]

- 1. **Chromatographic Conditions**

Mobile phase: acetonitrile (A)–0.2% phosphoric acid solution (B); injection volume: 1 μL; flow rate: 0.2 mL/min; column temperature: 30°C; detection wavelength: 265 nm. Gradient elution: 0–3 min, 5%→8% A; 3–15 min, 8%→15% A; 15–50 min, 15%→26% A; 50–60 min, 26%→42% A; 60–68 min, 42%→44% A; 68–73 min, 44%→70% A; 73–76 min, 70%→5% A; 76–80 min, 5% A; 60–68 min, 42%→44% A; 68–73 min, 44%→70% A; 73–76 min, 70%→5% A; and 76–80 min, 5% A.

- 1. **Preparation of Solutions**

### Preparation of the Test Solution

The appropriate amount of JT was obtained, weighed to 2 g, placed in a stoppered conical flask, supplemented with 20 mL of methanol, weighed, subjected to ultrasonic (power 250 W, frequency 45 kHz) treatment for 30 min, cooled and then weighed, with methanol used to compensate for the loss of mass. The solution was filtered with a 0.22 μm microporous filter membrane to produce the solution of the test material.

### Preparation of the Control Solution

Appropriate amounts of 5-Hydroxymethyl-2-furaldehyde, Salidroside, Chlorogenic acid , Cinnamic acid , Ammonium glycyrrhizinate , Rhein , Emodin , Clycyrrhetinic acid , Chrysophanol and Aloe-emodin controls were weighed precisely, and methanol was added to prepare a certain concentration of the control solution. An appropriate amount of each control was added to a volumetric flask, and methanol was added to prepare concentrations of 49.00, 103.50, 26.52, 18.96, 76.96, 25.97, 12.62, 9.68, 13.22, and 20.16 μg/mL, respectively.

- 1. **Drug Testing**

A Wayeal LC3600 series ultrahigh-performance liquid chromatograph (Anhui Wan Yi Technology Co., Ltd.) and a Luna® Omega Polar C_18_ column (150 mm×2.1 mm, 1.6 μm, Phenomenex, USA) were used. The 5-Hydroxymethyl-2-furaldehyde (Batch No. 111626-202316, purity: 98.4%), Salidroside (batch No. 110818-202210, Purity: 99.7%), Chlorogenic acid (batch No. 110753-202119, purity: 96.3%), Cinnamic acid (batch No. 110786-202305, purity: 98.8%), Ammonium glycyrrhizinate (batch No. 110731-202122, purity: 94.4%), Rhein (batch No. 110757-201607, purity: 96.0%), Emodin (batch No. 110756-201913, purity: 96.0%), Clycyrrhetinic acid (batch No. 110723-202316, purity: 99.6%), Chrysophanol (batch no. 110796-201922, purity: 99.4%), and Aloe-emodin (batch no. 110795-201710, purity: 98.3%) were purchased from the China Academy of Food and Drug Administration. Acetonitrile and phosphoric acid were chromatographically pure, and the water was ultrapure water.

# Results

- 1. **Methodological Examination**

One microliter each of the mixed control solution described in Section 1.2.2 and the test solution of JT described in Section 1.2.1 was added, and the mixture was injected into the ultrahigh performance liquid chromatograph according to the chromatographic conditions described in Section1.1. The chromatographic peaks and separation of 5-Hydroxymethyl-2-furaldehyde, Salidroside, Chlorogenic acid, Cinnamic acid, Ammonium glycyrrhizinate, Rhein, Emodin, Clycyrrhetinic Acid, Chrysophanol and Aloe-emodin were good, as shown in Figure 1.

|  |
| --- |
| Fig. 1: Methodological exclusivity diagram of JT. A: Mixed reference substances; B: Jingtian granule; 3. 5-Hydroxymethyl-2-furaldehyde; 5. Salidroside; 8. Chlorogenic acid; 15. Cinnamic acid; 19. Aloe-emodin; 20. Ammonium glycyrrhizinate; 21. Rhein; 23. Emodin; 24. Clycyrrhetinic Acid; 25. Chrysophanol |

- 1. **Precision Test**

JT (S11) was used to prepare the test solution according to Section 2.2.1, and the sample was injected continuously 6 times under the chromatographic conditions described in Section 2.1. The shape of peak No. 15 (cinnamic acid) in the chromatogram was better, and the response value was high; therefore, it was set as the reference peak, and the relative retention time and relative peak area of each common peak were calculated to be less than 2.9%, indicating that the precision of the instrument was good. The RSD of the relative retention time and relative peak area of each common peak was calculated to be <2.9%, indicating that the precision of the instrument was good.

- 1. **Stability Tests**

JT (S11) was used to prepare the test mixture according to Section 2.2.1. The sample was then injected for determination at 0, 4, 8, 12, 16, 20 and 24 h under chromatographic conditions described in Section 2.1. Peak 15 (cinnamic acid) was used as the reference peak, and the relative retention time and relative peak area of each common peak were calculated. The RSD of the relative retention time and relative peak area of each common peak was <2.4%, indicating that the test solution was stable for 24 h. The relative retention time and relative peak area of each common peak were <2.4%.

- 1. **Repeatability tests**

JT (S11) was used to prepare 6 test solutions according to the preparation method described in Section 2.1.1. Determination was performed under the chromatographic conditions described in Section 2.1. Peak 15 (cinnamic acid) was used as the reference peak, and the relative retention time and relative peak area of each common peak were calculated. The relative retention time and relative peak area of each common peak were calculated, and the RSD of each peak was less than 2.7%, which indicated that the reproducibility of the method was good.

- 1. **Establishment of Fingerprints of JT Particles**

The UPLC chromatograms of the 13 batches of samples were obtained by preparing test solutions of the 13 batches of samples according to the method described in Section 2.1.1, injecting the samples according to the chromatographic conditions described in Section 2.1, and then importing them into the software "Fingerprint Evaluation System of Traditional Chinese Medicine Chromatograms (2012 version)" for analysis and evaluation. The UPLC chromatograms of the 13 batches of samples were obtained and imported into the software "Chinese Medicine Chromatographic Fingerprint Similarity Evaluation System (2012 version)" for analysis and evaluation. The chromatogram of sample S13 was set as the reference chromatogram, with a time window of 0.1, and the median method was adopted, combined with multipoint calibration and marker peak matching, to generate the control chromatogram of JT granules and the UPLC superposition chromatogram of the 13 batches of samples (Figure 2). Twenty-five common peaks were identified in the control fingerprints, and 10 of them were recognized after matching with the control, which were 3. 5-Hydroxymethyl-2-furaldehyde;5. Salidroside; 8. Chlorogenic acid; 15. Cinnamic acid; 19. Aloe-emodin; 20. Ammonium glycyrrhizinate; 21. Rhein; 23. Emodin; 24. Clycyrrhetinic Acid; 25. Chrysophanol. The similarity of the fingerprint profiles was above 0.9, and the similarity of the profiles of the JT granules from the 13 batches was 0.985, 0.993, 0.992, 0.996, 0.983, 0.989, 0.955, 0.994, 0.978, 0.980, 0.994, 0.995, and 0.995, which indicated that the samples from each batch were highly similar to each other and that the process used to prepare the JT granules from each batch was relatively stable. The granule preparation process was relatively stable, and the quality consistency was good.

| 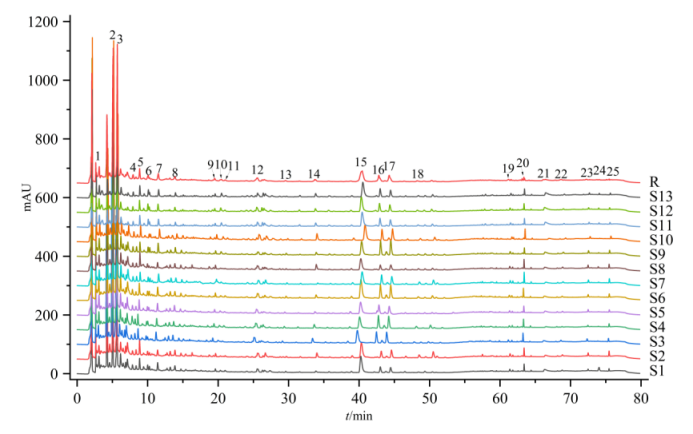 |
| --- |
| Fig. 2: fingerprints analysis of 13 batches JT (S1–S13: samples of JT; R: control fingerprints) |
